# Motivation Matters: Elucidating Factors Driving Exercise in People With Parkinson Disease

**DOI:** 10.1093/ptj/pzaf048

**Published:** 2025-04-10

**Authors:** Caro I Cools, Sonja A Kotz, Bastiaan R Bloem, Annelien A Duits, Nienke M de Vries

**Affiliations:** Faculty of Psychology and Neuroscience, Department of Neuropsychology & Psychopharmacology, Maastricht University, Maastricht, The Netherlands; Faculty of Psychology and Neuroscience, Department of Neuropsychology & Psychopharmacology, Maastricht University, Maastricht, The Netherlands; Department of Neurology, Centre of Expertise for Parkinson and Movement Disorders, Radboud University Medical Centre, Donders Institute for Brain, Cognition and Behaviour, Nijmegen, The Netherlands; Department of Psychiatry and Neuropsychology, Faculty of Health, Medicine and Life Sciences, School for Mental Health and Neuroscience, Maastricht University, Maastricht, The Netherlands; Department of Medical Psychology, Maastricht University Medical Center, Maastricht, The Netherlands; Department of Medical Psychology, Radboud University Medical Center, Nijmegen, The Netherlands; Department of Neurology, Centre of Expertise for Parkinson and Movement Disorders, Radboud University Medical Centre, Donders Institute for Brain, Cognition and Behaviour, Nijmegen, The Netherlands

**Keywords:** Depression, Exercise Adherence, Happiness, Interventions, Motivation, Parkinson Disease, Self-compassion

## Abstract

**Objective:**

Despite the known benefits of exercise for people with Parkinson disease (PwP), activity levels are not sustained over time due to various motivators and barriers impacting exercise adherence. Previously, studies on exercise adherence in PwP explored such barriers without describing or providing specific questions related to the determinants of motivation. Exercise adherence in PwP can be improved by addressing 3 key perspectives on motivation: personal factors (age, sex, premorbid motivation level, time when PwP started exercising, exercise before diagnosis, self-compassion), disease-related factors (perceived disease severity, depression score), and environmental factors (distance to exercise therapy, weather conditions, encouragement received from partners).

**Methods:**

Six hundred seventy-two PwP from the Netherlands participated in an online survey that comprised questionnaires on demographics, depression, self-compassion, perceived disease severity, and additional questions on sports, motivation, and barriers related to sports. A multiple regression analysis was applied with current motivation as an outcome measure, and age, sex, perceived disease severity, premorbid motivation, depression, self-compassion, age starting exercising, and exercise before diagnosis as determinants.

**Results:**

The results revealed that current motivation levels to exercise are associated with higher levels of premorbid motivation (*b* = 0.14), greater self-compassion (*b* = 0.32), lower age (*b* = −0.03), lower perceived disease severity (*b* = −0.10), and lesser degrees of depression (*b* = −0.10). Barriers stopping PwP from exercising were fatigue, weather conditions, and having less energy for other activities after exercising.

**Conclusion:**

Understanding these motivational factors and barriers helps shape and promote better exercise adherence and thereby ascertain greater symptomatic benefits for PwP.

**Impact:**

This study outcome gives health care professionals insight into determinants of motivation and exercise adherence, which will help enabling tailored approaches for improved engagement.

## INTRODUCTION

Current treatment for Parkinson disease (PD) focuses on improving or stabilizing both non- and motor symptoms. As pharmacological interventions mainly focus on motor symptoms, additional non-pharmacological interventions are necessary.^1^^,^^2^ One of these interventions encompasses exercising. Increasing evidence shows that exercise ameliorates both motor (ie, gait, balance) and non-motor symptoms such as depression, sleep, or cognitive problems.^3^ In turn, any improvement in motor^1^ and non-motor symptoms^4^ may have a positive impact on daily life activities, quality of life, self-esteem, and happiness.^5^

Despite these benefits of exercise, 1 study shows that only 27% of people with Parkinson disease (PwP) appeared to meet the recommended guidelines for physical activity.^6^ Levels of physical activity of PwP are, in general, not sustained over time.^7^ Compared to the general population, PwP are less active even during the early disease stage.^2^ Fifty percent of participants drop out from exercise programs in the first 6 months of a program.^8^ Consequently, it is urgent to investigate what could motivate PwP better to engage in and continue exercising as self-determined motivation can significantly explain 12% to 16% of the variance in exercise adherence when measured 4 weeks into an intervention.^9^

A comprehensive review^10^ listed several exercise barriers and motivators in PwP, revealing important factors influencing exercise adherence in both clinical trials and everyday life. Among the barriers were low expectations of benefits, severe motor symptoms, lack of time, fear of falling, apathy, and fatigue. Conversely, enjoyable exercises, a supportive environment,^11^ and training in pairs during exercise classes^12^ seem to be important motivators for starting and adhering exercises. Other known positive inducers on exercise behavior are higher education, older age,^12^^,^^13^ support from others such as family, and encouragement and feedback from health care professionals for feelings of safety and a sense of community among the people involved.^12^

Motivation derives from “to move,” and when someone is motivated, it implies that this person is energized or activated toward an end.^14^ Motivation is an important reason for engaging in physical activities as it is defined as the force that energizes, drives, and directs behavior.^15^

Motivation directly contributes to well-being and rehabilitation outcome effects.^16^ The self-determination theory describes intrinsic and extrinsic motivation through behavioral change.^14^ Intrinsic motivation is the motivation a person derives from oneself. A systematic review by Eynon and colleagues^17^ showed that intrinsic motivation is a key factor associated with exercise adherence in multiple studies. Often, intrinsic motivation is more sustainable than extrinsic motivation as it is self-induced and independent of external pressures.

Extrinsic motivation can either be reflected as externally controlled or doing something because it leads to a separable outcome.^14^ Extrinsic motivation often plays a role when starting exercising as people might conceive the associated benefits of exercising as expressed by health care professionals.^17^

While physical activity is linked to actual behavior and can be clearly measured, motivation reflects the underlying psychological drivers that initiate and sustain such behavior. Therefore, though difficult to measure, our working definition for motivation is “being energized to engage in exercise” and this is consequently the outcome measure.

A direct regulator of motivation is dopamine,^18^ which is depleted in PwP,^19^ and can affect motivation negatively.^18^ Verbal reports and interviews are often used to better grasp subjective measures of motivation. Further, past behavior is a strong forecaster of future exercise adherence.^20^ Given the challenges in assessment, motivation is hardly ever included in PD research as an outcome or mediating factor of exercise adherence.

So far, exercise adherence studies in PwP have focused on outlining barriers and motivators, without including specific questions about the determinants of motivation. As motivation is mostly studied in a population that is healthy,^20^ direct investigation of experienced motivators and barriers in PwP is often missing in exercise research. Understanding motivation is crucial, as it can inform about adherence to exercise interventions. Therefore, this research aimed to identify factors influencing motivation to exercise in PwP. The categories from the comprehensive review on motivation^10^ were adjusted based on the factors explored in this study. Hence, we defined 3 categories of determinants of motivation: personal factors (eg, age, sex, premorbid motivation level, the age at which PwP started exercising, exercise before diagnosis, self-compassion to measure happiness^21^), disease-related factors (eg, perceived disease severity, depression score), and environmental factors (eg, distance to exercise therapy, weather conditions, encouragement received from partners).

We formulated the following hypotheses: age is affecting current motivation as it also significantly predicts exercise adherence^8^ and older age is associated with increased exercise likelihood.^13^ Research further showed that exercise preferences differ between sex,^2^ suggesting such differences might show in PD. We further expected to replicate findings of increased self-compassion leading to higher motivation levels.^16^

Previous motivation as well as the amount of exercise before diagnosis were expected to positively influence motivation levels as indicated by research on future exercise adherence in people who are healthy.^20^ The age by which exercising started was previously not investigated in PwP, making it difficult to generate hypotheses about their influence on current motivation levels. It could be argued that the age when exercising started relates to the degree of exercising before diagnosis, which should influence motivation.^20^

Since self-compassion and depression are related, it is expected that depression negatively affects motivation levels.^16^ No prior studies, involving PD or other neurodegenerative diseases, investigated the relation between disease severity and motivation. Higher depression scores are associated with disease severity and motor complications,^22^ but the onset of depressive syndromes do not necessarily parallel the evolution of motor dysfunctions.^23^ Due to the direct connection between depression and disease severity,^22^ we expect enhanced perceived disease severity influencing motivation indirectly via depression and therefore affect motivation levels negatively.

## METHODS

We performed a cross-sectional study, using an online survey. The survey consisted of a combination of several validated questionnaires on depression, self-compassion, physical activity during the last 2 weeks, and additional questions on perceived disease severity, sports, and motivation and barriers for exercise.

### Participants

Invitations to participate were distributed via multiple channels. As one of many items in newsletters, it is unclear how many participants opened or read the newsletter and proceeded to click on the survey invitation. Some people received a link to the survey multiple times as they subscribe to different newsletters. Non-PwP might also have received survey invitations. We included PwP based on self-reported PD diagnosis, excluding self-reported forms of atypical Parkinsonism. No other criteria have been applied. Ultimately, 719 people responded to the survey.

### Procedure and Survey

The online survey was sent out between November 2021 and March 2022 through multiple channels: ParkinsonNEXT, an online platform where PwP and their peers can participate in research projects; the website and newsletters of the Dutch Parkinson Society; and newsletters of the Parkinson Cafés (monthly get-togethers for PwP and peers) in 4 cities in the Netherlands. Participants were invited to participate anonymously, and no personal, identifiable information (including date of birth, date of diagnosis, place of residence, IP addresses) was collected. The study adhered to the Declaration of Helsinki and did not require additional ethical approval as it is anonymous and does not contain any compromising information. The online survey took 30 to 45 minutes to complete, and participants could stop at any time.

The online survey consisted of a combination of open and closed questions and included several validated questionnaires or a number of items of specific questionnaires (see Suppl. Material S1 for an overview). It started with some demographic questions (items 1.1–1.13, 1.20, 1.21) and general questions about perceived disease severity (items 1.14–1.19), followed by creating a top 3 out of 19 motivators and 27 barriers that were previously identified as important for PwP (see Suppl. Material S1 for an overview of motivators and barriers).^10^ The survey included personal, disease-related, and environmental factors that might influence the motivation to exercise. The current self-perceived motivation level was measured by asking participants to rate their motivation on a scale from 1 to 10, with 10 indicating a high level of motivation (item 3.1).

Personal factors included premorbid motivation levels (item 3.2), measured on a similar 10-point scale, exercise history (items 2.1–2.3), and self-compassion (Self-Compassion Scale)^24^ for happiness.^21^ Disease-related contributors included depression scores (the depression questions from the Hospital Anxiety and Depression Scale)^25^ and perceived disease severity. Perceived disease severity was measured by evaluating balance (item 1.14), walking in general (item 1.15), freezing (item 1.16), walking 1 km (item 1.17), walking 100 m (item 1.18), and holding a cup (item 1.19). Item 1.16 was based on the Freezing of Gait scale (FOG),^26^ items 1.17–1.19 are based on items of the mobility dimension of the Parkinson’s Disease Quality of Life Questionnaire (PDQ-39)^27^ and items 1.14 and 1.15 are not derived from a previously validated questionnaire. Together, these items resulted in a minimum and maximum score, respectively, 6 to 29, with higher scores indicating more severe PD. Environmental factors included distance to exercise therapy, weather conditions, and support received from partners.

For survey data to be included in the analyses, answers to questions about and including sport preferences were mandatory. Surveys missing these were considered insufficient and were excluded.

### Data Analysis

The data were analyzed using IBM SPSS Statistics (IBM Corp., Armonk, NY, USA).^28^ First, total scores of perceived disease severity (items 1.14–1.19), depression (based on the depression questions from the Hospital Anxiety and Despression Scale [HADS]),^25^ and well-being^24^ were calculated. Identification of most important barriers and motivators was done by asking participants to rank them as the top 3, and via questions regarding the influence of partners and friends on motivation for exercising.

To identify personal and disease related factors that may impact motivation, a multiple regression analysis was applied with current motivation level as the outcome and age, sex, perceived disease severity, premorbid motivation level, total score of depression, total score of self-compassion, exercise before diagnosis, and age starting exercising as determinants. An alpha value of .05 was used to determine statistical significance.

## RESULTS

A total of 719 people responded to the online survey, with 47 participants excluded from further analysis (*n* = 25 completed fewer than the mandatory number of questions; *n* = 22 not diagnosed with PD). The final sample size was 672 participants, consisting of 577 completed surveys and 95 incomplete surveys (PwP completed more than the mandatory number of questions, up to and including the questions regarding sports, and these answers were therefore included in the analysis).

On average, participants were 65.68 years old (SD = 8.24; range 38–88 years) and 14.1% (*n* = 97) were 50 years or younger at the time of diagnosis. The perceived disease severity had a mean of 9.82 (SD = 3.87; min = 6, max = 25). The respondents consisted primarily of exercisers, with only 6.5% currently not engaging in any type of exercise. In the final sample, 19.3% were involved in regular exercises as well as PD-specific exercises. All sociodemographic, clinical characteristics, and exercise characteristics of the participants are reported in Table 1.

**Table 1:** Sociodemographic, Clinical, and Exercise Characteristics of Participants^*a*^

**Characteristic**	**Participants (*n* = 672)**
Age, year, mean (SD)^*b*^	65.68 (8.24)
Sex, *n* (%)^*c*^	
Male	384 (57.2%)
Female	286 (42.6%)
Other	1 (0.1%)
Disease duration (symptom since years), *n* (SD)^*d*^	9.51 (6.36)
Disease duration (diagnosis since years), *n* (SD)^*b*^	6.68 (5.05)
Perceived disease severity (min = 6; max = 25)	9.82 (3.87)
Symptoms, *n* (%)	
Left	258 (38.4%)
Right	245 (36.5%)
Both sides	169 (25.1%)
Tremor, yes, *n* (%)	382 (56.8%)
On medication, yes, *n* (%)	632 (94.0%)
Dopamine/apomorphine pump, yes, *n* (%)	29 (4.3%)
DBS, yes, *n* (%)	58 (8.6%)
Motivation levels for exercise before diagnosis, *n* (SD)^*e*^	7.45 (1.81)
Motivation levels for exercise now, *n* (SD)^*e*^	7.99 (1.77)
Non-specific PD exercises, yes, *n* (%)	628 (93.6%)
Specific PD exercises, yes, *n* (%)	130 (19.3%)
Age starting exercising, years, *n* (%)^*f*^	
Never	33 (5.0%)
<10	282 (42.6%)
10–20	159 (24.0%)
20–30	75 (11.3%)
>30	113 (17.1%)
Since diagnosis, amount of exercise, *n* (%)	
Remained unchanged	132 (19.9%)
Decreased	245 (37.0%)
Increased	285 (43.1%)
Energy decreased because of Parkinson disease, yes, *n* (%)	527 (78.4%)
Energy loss causing a decrease in exercise, yes, *n* (%)^*g*^	254 (48.2%)
Performs exercise … per week before diagnosis, *n* (%)^*f*^	
I was not physically active	14 (2.1%)
I was physically active a few times a month	20 (3.0%)
I was physically active 1× a week	34 (5.1%)
I was physically active 2× a week	124 (18.7%)
I was physically active 3× a week	16 (2.4%)
I was physically active 4× a week	5 (0.8%)
I was physically active 5× a week	4 (0.6%)
I was physically active 6× a week	1 (0.2%)
I was physically active every day	292 (44.1%)
I was physically active multiple times a day	129 (19.5%)
Other option…^*h*^	23 (3.5%)
More motivated when you self chooses the exercise, yes, *n* (%)^*i*^	105 (86.8%)

^*a*^DBS = deep brain stimulation.

^b^ *n* = 670.

c *n* = 671.

d *n* = 651.

e *n* = 661.

f *n* = 662.

g *n* = 527.

h Other option consisted of 8 people (1.2%) mentioning being physically active “three to four or five times a week” or “several times a week.” Four people gave unrelated answers regarding their work (“always procrastinating, work, work, and work,” “was always busy,” “work with a lot of movement,” “just worked”), 2 people checked other option but did not fill anything in at all, and 9 people just mentioned different exercises without specifying how often they were physical active.

i *n* = 121.

### Perceived Motivators and Barriers

In the online survey, PwP rated the most influential motivators and barriers for exercising as seen in Table 2.

**Table 2:** Three most Important Motivators and Barriers

**Motivators**	**%**	**Participants (*n* = 672)**
I would like to continue to function independently for as long as possible	57.4	386
I notice a positive effect of exercise on overall health	47.2	317
I notice a positive effect of exercise on Parkinson’s symptoms	37.8	254
**Barriers**	%	
Fatigue hinders me	35.7	240
The weather conditions hinder me	19.5	131
I have less energy for other activities after exercise	17.4	117

### Determinants on Current Motivation: Personal and Disease-Related Factors

The current motivation level of PwP to exercise was positively associated with their premorbid level of motivation (*b* = 0.15; *P* < .001,) and self-compassion (*b* = 0.32; *P* = .016). The current motivation level was negatively associated with age (*b* = −0.03; *P* = .002), depression (*b* = −0.10; *P* < .001), and perceived disease severity (*b* = −0.10; *P* < .001). See for more information Table 3. Higher age, depression, and perceived disease severity scores were associated with lower motivational scores. In total these 5 factors explained 20% of the variance in relation to current motivation. The determinant sex was not significantly associated with current motivation level (*P* > .15). The Figure 1 summarizes the main results.

**Table 3:** Determinants on Current Motivation*^a^*

**Factor**	**Unstandardized Coefficients (*b*)**	**Standardized Coefficients (Beta)**	**Confidence Interval (95%)**	**Sign. (*P*)**
Premorbid motivation	0.15	.15	0.07 to 0.22	<.001
Self-compassion	0.32	.11	0.06 to 0.58	.016
Age	−0.03	−.12	−0.04 to −0.01	.002
Depression	−0.10	−.20	−0.14 to −0.05	<.001
Perceived disease severity	−0.10	−.21	−0.14 to −0.06	<.001
Sex	0.19	.06	−0.07 to 0.45	>.15

^a^ Sign. = .05.

In an explorative analysis, the factors, age starting exercising and exercise before diagnosis, were added to the model. Neither of them led to changes in the original model nor revealed a significant association with the original model (*P* > .40).

Several motivational reasons for choosing various exercises were offered to PwP. For each exercise they preferred, participants had to argue their reason of motivation. The biggest motivator was “I have participated in this kind of exercise before” (76.8%). Table 4 gives a percentual overview regarding the motivational reasons behind participating in exercises.

**Table 4:** Motivational Reasons for Exercise

**Why Are You Interested in Participating in This Exercise?**	**%**
I have participated in this exercise before	76.8
I would like to try out new exercises	39.6
Other people I know have participated in this exercise before and were enthusiastic about it	15.8
I have participated in this exercise, research based	0

### Determinants on Current Motivation: Environmental Factors

Regarding the most influential barriers, weather was rated at 19.5%. Another environmental factor was exercising with a partner, which can be considered as an external motivating factor. From all participants, 7.7% participated in studies focusing on exercising for PwP before. Of these participants, 28.8% joined the exercise study together with a partner. When surveying for future exercise interests, 15.8% participants indicated increased interest in exercise because friends and partners were thinking positively about that chosen exercise.

## DISCUSSION

Most studies on exercise adherence in PwP overview barriers and motivators, without providing specific questions related to them. This study focused on the determinants of motivation to exercise from a personal, disease-related, and environmental perspective. Results indicated that higher current motivation levels were associated with higher premorbid levels of motivation, greater self-compassion, lower age, lower perceived disease severity, and less depression. We discuss the relevance of these factors in turn.

### Personal and Disease-Related Factors

Previously, motivational patterns and exercise adherence were only studied in participants who are healthy.^20^ Here we replicated these objectives in PwP, confirming the impact of motivation to exercise in PwP. We found a significant association between motivation and personal and disease-related factors, with positive associations between motivation and premorbid motivation and self-compassion levels and negative associations between motivation and age, perceived disease severity, and depression. These 5 factors explained 20% of the variance related to current levels of motivation to exercise.

However, we found age to be associated with increased current motivation, leading to the opposite association in PwP than in previous studies with participants who are healthy.^8^^,^^13^ This might relate to age not being a direct mediator of motivation, but that diagnosis time and perceived disease severity might impact PwP motivation to exercise.

Sex, exercise before diagnosis, or the age starting exercising were not associated with current motivation. Not replicating the findings of exercise before diagnosis^20^ within a PD population is surprising, especially given the body of evidence from exercisers who were healthy. This finding underscores the importance of exploring PD-specific factors influencing motivation. Unfortunately, this finding does not allow any conclusions regarding motivation to exercise before diagnosis. Possible explanations are PD-specific factors disrupting the association of exercise before diagnosis with current motivation. Looking at the data of exercise before diagnosis, 63.6% of PwP exercised once or multiple times a day or every day. This potentially limits the variability of this variable and subsequently may have masked a potential association with motivation. The age when starting exercising as well as sex were not previously investigated in relation to the motivation to exercise in PwP, making it difficult to predict any effects on motivation.

### Environmental Factors

15.8% of PwP were motivated to try certain exercise interventions based on positive recommendations of friends and family. With a 5-option question regarding motivation, the main motivator (62.3%) was having “previous experience in joining the chosen exercise.” This also confirmed the finding of positive impact of past behavior on future exercise adherence.^20^ With each PwP having a different and unique background, differences in exercise adherence are not surprising. This past behavior is another indicator favoring an individualized exercise intervention rather than a one-size-fits-all approach. This main motivator furthermore relates to the importance of relatedness and experiencing autonomy, 2 factors involved in intrinsic motivation according to the self-determination theory.^8^ Furthermore, only the barrier weather conditions (19.5%) could be dedicated to environmental influences, replicating a previous finding.^10^

### Study Limitations

While confirming several hypotheses, the current study also shows some shortcomings. One limitation is the relatively low response rate given the number of invitations sent. It remains unclear how many unique PwP were approached as it is unclear how many PwP received similar newsletters multiple times or opened them without taking any further action. Consequently, we could not define a response rate a priori.

Of the respondents, 93.6% were exercising, introducing a selection bias in favor of exercising, likely not representing the general views on exercise interventions. This might imply an already higher adherence and higher motivation in this group of PwP, compared to the general population. On the other hand, this sample’s high exercising rate offers the opportunity to identify the determinants of their motivation to do so.

This selection bias also occurred in the factor exercise before diagnosing, possibly masking an association with current motivation levels. To avoid this in future research, one should strive to recruit a more heterogeneous cohort regarding factors associated with motivation.

Relying on self-reported information rather than any clinical measure used by neurologists such as the Hoehn and Yahr stages,^29^ or the Movement Disorders Society Unified Parkinson Disease Rating Scale (MDS-UPDRS),^30^ renders conclusions about this study regarding perceived disease severity and can be difficult compared with other research. Motivation, on the other hand, is a subjective concept and the question is whether PwP feel limited or motivated by their perceived disease severity.

### Implications for Future Research

As shown in the Figure, several determinants were not investigated here, holding implications for future research. Although participants in this study were likely more exercise-prone compared to PwP in general, the factors associated with motivation did not fully explain adherence to exercise. With only 20% of variance being explained, we acknowledge that other factors might play a role as well. Future research should focus on exploring these possible contributing factors, such as differences in motor symptoms, and the importance of psycho-education for motivation and exercise adherence. As motivation reflects the drive that initiates and sustains actual behavior, it might be interesting to take a closer look at the association between motivation levels and physical activity of PwP. Differences in motivation between high- and low-exercisers^11^ could be an important influencing component, as high-exercisers were more likely to start exercising after being diagnosed. Low-exercisers were more likely to reduce the amount of exercise and require more motivating factors to adhere to.^11^

**Figure:**
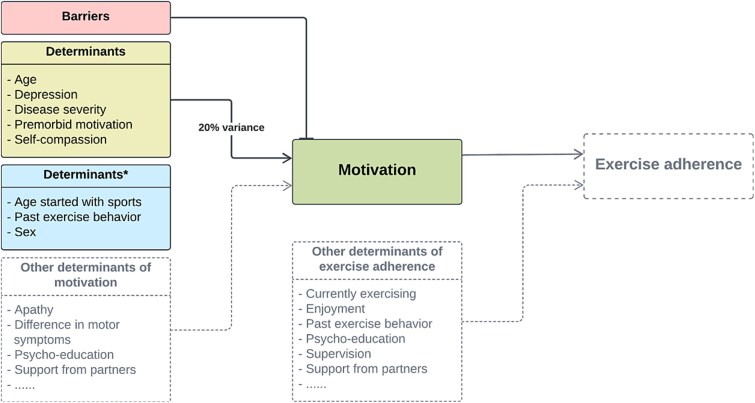
Determinants included in this research are presented in the colored boxes. Solid lines represent determinants associated with motivation. No line and marked with the ^*^ represent insignificant results for these determinants per this research. The dotted lines in the figure represent determinants that were not studied within this research, holding implications for future research. For an elaborated overview of the barriers, see Supplementary Material S1.

Future research should create a better understanding of what environmental factors entail, especially as they can influence whether PwP continue to exercise or not. Most barriers linked to environmental factors could be easily addressed (eg, by providing exercise at home when the weather is bad or providing transportation options).^10^

For future research, it might be interesting to look into motivational differences within the PD subtypes. The postural instability and gait disorder subtype is being linked to reduced dopaminergic levels as opposed to the tremor dominant subtype.^31^ The dopamine-driven subtype might therefore experience generally less motivation. Another interesting determinant that future studies should include, is apathy. Apathy is part of the disorders of motivation and is common in PD.^18^ Given that rehabilitation research shows apathy questionnaires serve as a useful tool assessing apathy and motivation, it could offer opportunities to specify the connection between apathy, motivation, and exercise adherence.

Regarding the influence of disease severity on motivation, it will be meaningful to replicate findings to determine its significance to motivation. Importantly by quantifying disease severity by either the use of the MDS-UPDRS^30^ or by using the Hoehn and Yahr scale.^29^ Our study design did not allow for causal inferences regarding disease severity, as no variable was controlled by the design. Importantly, future research should investigate whether disease severity has a direct or indirect effect on motivation. Possible moderating effects, initiating those indirect effects, would be self-compassion or depression, as they both influence motivation and are related to disease severity, although inconsistently within the literature.

### Clinical Implications

Understanding exercise adherence in PwP involves identifying key motivational factors, for both being and staying motivated. Such insight assists health care professionals, partners, and PwP in improving exercise adherence. This study highlights the impact of premorbid motivation, self-compassion, age, depression, and perceived disease severity on motivation. Suggesting early initiation of exercise being crucial as motivation tends to be higher in younger PwP and those with lower disease severity. Giving PwP with higher depression ongoing support to sustain their exercise routines is essential, as depression negatively impacts motivation to adhere to exercise. These insights impact the way to effectively approach PwP to persuade them to exercise.

Pinpointing areas needing more exploration to improve exercise adherence and removing barriers for exercising is essential in creating higher levels of motivation. This includes addressing fluctuating energy levels and fatigue during exercises. This uncovering of contributing factors to motivation marks an initial phase from which better clinical guidance can start for PwP.

## CONCLUSIONS

We showed higher current motivation levels to exercise being associated with higher premorbid levels of motivation, greater self-compassion, lower age, lower perceived disease severity, and less depression. As it remains difficult to conclude which determinants fully drive motivation, we consider this as the first step to identify and consolidate motivational determinants.

## Supplementary Material

2024-0505_R1_Supplementary_Material_pzaf048

## Data Availability

Data will be made available upon request via a link.
