# Supplementary material for: Motivation Matters: Elucidating Factors Driving Exercise in People With Parkinson Disease
Source: Phys Ther. 2025 Apr 10;105(6):pzaf048. doi: 10.1093/ptj/pzaf048 (PMC12131318; doi:10.1093/ptj/pzaf048)
Supplement: 2024-0505_R1_Supplementary_Material_pzaf048 [file 2024-0505_r1_supplementary_material_pzaf048.pdf]

Supplementary Material
Translated version (Dutch to English) of the online survey questions

1. General Questions **(not derived from an official questionnaire)**

1.1 What is your age?

1.2 What is your sex?
0 Male
0 Female

1.3 What is your nationality?
0 Dutch
0 Belgian
0 Other, namely ....

1.4 What is your marital status?
0 Married/ living together
0 Partnered, but not living together
0 Single
0 Widowed

1.5 What is your highest completed education?
0 Primary education
0 Lower vocational education (LBO, MAVO, VMBO)
0 HAVO
0 VWO (atheneum, gymnasium, lyceum)
0 Secondary vocational education (MBO)
0 Higher vocational education (HBO)
0 University (WO)
0 None of these (less than 6 years of primary education)

1.6 In which year were you diagnosed with Parkinson's disease?

1.7 In which year, looking back, did you first notice the initial symptoms?

1.8 Do you have symptoms on the left and/or right side?
0 Left side
0 Right side
0 Both left and right side

1.9 Do you have tremors?
0 Yes
0 No

1.10 Are you taking medication?
0 Yes

0 No

1.11 Do you have a dopamine/apomorphine pump?
0 Yes
0 No

1.12 Do you have a neurostimulator (DBS)?
0 Yes
0 No

1.13 Are you undergoing physiotherapy for Parkinson's disease?
0 Yes, individual sessions
0 Yes, through an exercise group
0 Yes, both individual sessions and an exercise group
0 No

1.14 Your balance is:
0 Good
0 Sometimes I need to grab onto something to avoid falling
0 I fall once a month
0 I fall once a week
0 I fall more than once a week

1.15 Your walking is:
0 Good, not affected
0 Affected, but I can still walk without aids
0 I need aids for walking
0 I can no longer walk

1.16 Do you feel like your feet are glued to the floor when you walk, turn, or when you start walking (freezing)? **(derived from FOG questionnaire)**
0 Never
0 Very occasionally, about once a month
0 Sometimes, about once a week
0 Often, about once a day
0 Always when you walk

1.17 Have you had problems walking 1km? **(derived from PDQ-39 questionnaire)**
0 Never
0 Occasionally
0 Sometimes
0 Often
0 Always

1.18 Have you had problems walking 100 meters? **(derived from PDQ-39 questionnaire)**

0 Never

0 Occasionally
0 Sometimes
0 Often
0 Always

1.19 Do you have problems holding a glass or cup without spilling? **(derived from PDQ-39 questionnaire)**

0 Never
0 Occasionally
0 Sometimes
0 Often
0 Always

1.20 Do you have less energy due to Parkinson's disease?
0 Yes
0 No

1.21 If yes, does the loss of energy result in less exercise or movement?
0 Yes
0 No

<u>2. Exercise History</u>
2.1 How often did you exercise before being diagnosed with Parkinson's disease? By exercise, we mean gardening, walking, cycling, and sports activities. **(based on work from another research group)**

0 I did not exercise
0 I exercised a few times a month
0 I exercised one time a week
0 I exercised two times a week
0 I exercised three times a week
0 I exercised four times a week
0 I exercised five times a week
0 I exercised six times a week
0 I exercised every day
0 I exercised multiple times a day
0 Other, namely ....

2.2 From what age did you start doing sports?
0 I have never done sports
0 Since childhood (<10 years)
0 As a teenager (10-20 years old)
0 Between my 20s and 30s
0 After my 30s (>30 years old)

2.3 Has your exercise pattern changed since you were diagnosed with Parkinson's disease?

0 My exercise pattern has not changed
0 I exercise less after the diagnosis
0 I exercise more after the diagnosis

3. General Impression of Motivation to Exercise
3.1 On a scale of 1 to 10, how motivated are you currently to exercise?

3.2 On a scale of 1 to 10, how motivated were you to exercise before your diagnosis?

4. Motivations to exercise: **(based on work from another research group)**

1. I exercise because I notice a positive effect on overall health.
2. I exercise because I have high expectations of its effects on overall health.
3. I exercise because I notice a positive effect on Parkinson's symptoms.
4. I exercise because I have high expectations of its effects on Parkinson's symptoms.
5. I exercise because I want to remain independent for as long as possible.
6. Exercising has a positive effect on my mood.
7. Exercising has a positive effect on my mental health.
8. I have sporting goals that I want to achieve.
9. Exercising gives me more confidence in my body.
10. Exercising gives me a good feeling afterward.
11. Social contact with others during exercise is important to me.
12. I enjoy competition.
13. My friends and/or family encourage me to do so.
14. My doctor or therapist advises it.
15. There are many opportunities in my environment to exercise and play sports.
16. I feel guilty if I don't exercise.
17. I can easily incorporate exercise into my daily life.
18. I have a tailored exercise program.
19. Because I enjoy it.

5. Barriers to exercise: **(based on work from another research group)**

1. I find it difficult to get moving because I don't notice a positive effect on overall health.
2. I have low expectations of the effects of exercise on overall health.
3. I don't notice a positive effect of exercise on Parkinson's symptoms.
4. I have low expectations of the effects of exercise on Parkinson's symptoms.
5. Health problems (not related to Parkinson's disease) hinder me.
6. Problems with movement related to Parkinson's disease (such as balance issues) hinder me.
7. I am afraid of falling.
8. Fatigue hinders me.
9. Anxiety hinders me.
10. Depression hinders me.
11. I have less energy for other activities after exercising.
12. My medication hinders me.
13. Shame about my limitations hinders me.

14. I am confronted with people with Parkinson's disease in an advanced stage during exercise.
15. I feel intimidated by other athletes.
16. Disappointment in my own movement hinders me.
17. I have little interest in exercise.
18. I do not take the initiative to exercise.
19. I believe I already get enough exercise in my daily life.
20. Lack of encouragement from my therapist/doctor hinders me from exercising.
21. My doctor/therapist does not attach much importance to exercise.
22. Weather conditions hinder me.
23. My living environment does not appeal to me/is not suitable (e.g., uneven sidewalks, sports facilities too far away, no park nearby).
24. I don't have time for it.
25. I don't have enough knowledge about exercising with Parkinson's disease.
26. I don't have buddies to exercise with.
27. I cannot afford it.

6. HADS to measure depression

7. SCS to measure self-compassion and happiness
